# Serum-derived exosomal PD-L1 expression to predict anti-PD-1 response and in patients with non-small cell lung cancer

**DOI:** 10.1038/s41598-021-87575-3

**Published:** 2021-04-09

**Authors:** Yoshihisa Shimada, Jun Matsubayashi, Yujin Kudo, Sachio Maehara, Susumu Takeuchi, Masaru Hagiwara, Masatoshi Kakihana, Tatsuo Ohira, Toshitaka Nagao, Norihiko Ikeda

**Affiliations:** 1 Department of Surgery, Tokyo Medical University Hospital, 6-7-1 Nishishinjuku, Shinjuku-ku, Tokyo, 160-0023 Japan; 2 Department of Anatomical Pathology, Tokyo Medical University Hospital, Tokyo, Japan

**Keywords:** Biomarkers, Medical research

## Abstract

PD-L1 expression is the most useful predictive biomarker for immunotherapy efficacy on non-small cell lung cancer (NSCLC), and CD8+ tumor-infiltrating lymphocytes (CD8+ TILs) play an essential role in the clinical activity of immunotherapy. PD-L1 is found on the exosome’s surface, and PD-L1 expressing exosomes can inhibit antitumor immune responses. This study aimed to analyze tumor PD-L1 expression, serum exosomal PD-L1, and CD8+ TILs to investigate anti-PD-1 response and clinicopathological outcomes in NSCLC. One hundred twenty patients with stage I–III NSCLC were enrolled, and serum samples collected during the initial surgery were pooled. The Human CD274/PD-L1 ELISA kit was used to quantify the exosomal PD-L1. Exosomal PD-L1 levels were significantly correlated with tumor PD-L1 levels (p < 0.001) and the number of CD8+ TILs (p = 0.001). Patients with exosomal PD-L1 ≥ 166 pg/mL tended to have a worse RFS than those with < 166 pg/mL in all stage (p = 0.163) and stage I patients (p = 0.116). Seventeen patients exhibited postoperative recurrences and received anti-PD-1 treatment. The disease control rate of patients with exosomal PD-L1 ≥ 166 pg/mL was 100%. The measurement of serum exosomal PD-L1 as a quantitative factor with tumor PD-L1 status may help predict anti-PD-1 response and clinical outcomes in patients with NSCLC.

## Introduction

The advent of immune checkpoint inhibitors (ICIs), specifically of antibodies targeting the programmed cell death-1 (PD-1)/programmed cell death ligand 1 (PD-L1) pathways, has revolutionized treatment for non-small cell lung cancer (NSCLC)^1–5^. PD-L1 immunohistochemical expression is currently the most useful biomarker correlating with immunotherapy efficacy for NSCLC^1–4,6^. Pre-existing anti-tumor immunity such as the presence of CD8^+^ tumor-infiltrating lymphocytes (CD8+ TILs), has also been reported to play an essential role in the activity of anti-PD-1/PD-L1 immunotherapy and in predicting therapeutic efficacy^7–9^.

Exosomes, small membrane vesicles (30–150 nm) of endocytic origin, are known to act as intercellular messengers that can shuttle cargos, such as mRNA, proteins, miRNA, and lipids between cells^10–13^. They have been studied in cancer diagnostics as cancer-derived exosomes are involved in metastatic cascades, such as invasion, migration, and the priming of metastatic niches^14–17^. PD-L1 is found on the exosome' surface, and exosomal PD-L1 expression has been associated with both tumor progression and suppression of the anti-tumor immune response^18–21^. However, the clinical impact of exosomal PD-L1 and CD8+ TILs on the anti-PD-1/PD-L1 response and on survival in early-stage NSCLC remains unclear.

This study aimed to analyze the tumor microenvironment based on tumor PD-L1 expression, serum exosomal PD-L1, and CD8+ TILs to investigate the anti-PD-1 response and clinicopathological outcomes in NSCLC.

## Methods

### Study population

Between January 2015 and December 2016, 452 patients underwent pulmonary resection for primary lung cancer at the Tokyo Medical University Hospital. We included 363 patients who underwent complete anatomical resection (lobectomy or segmentectomy) for pathological stage I to III NSCLC and had invasive cancers excluding neuroendocrine cancers (Supplementary Fig. S1). Blood samples were taken from patients immediately before the initial surgery in the operating room. Preanalytical review of representative Hematoxylin and Eosin (H&E) sections on tumor and blood samples excluded 243 patients from 363 patients, and ultimately, 120 patients were enrolled in this study. TNM stage was determined in accordance with the 8th edition of the TNM Classification of Malignant Tumors. The Institutional Review Board of Tokyo Medical University (SH4064) approved this study. Informed consent for the use and analysis of clinical data was obtained preoperatively for each patient.

### Histopathology

After the tissue specimens were fixed with formalin and embedded in paraffin, serial 4-µm sections were stained with Hematoxylin and Eosin. All slides were evaluated by a pulmonary pathology specialist. Immunohistochemical (IHC) staining for PD-L1 (E1L3N, #113684; CST) and CD8 (M710301-2; Agilent) was performed on whole-section samples. Briefly, 5 µm thick formalin-fixed, paraffin-embedded tissue sections were deparaffinized and rehydrated. Antigen retrieval was performed using a tissue cooker containing 250 mL of citrate (pH 6.0). The slides were incubated with primary antibodies overnight, followed by horseradish peroxidase (HRP)-conjugated polymer secondary antibody (MAX-PO; Nichirei Biosciences) developed with chromogranin substrates and counterstained with hematoxylin. The stained slides were simultaneously evaluated by a pulmonary pathologist and a thoracic researcher; discrepancies were resolved by consensus. Each tumor section was evaluated for CD8+ TILs, and three independent areas with the most abundant TILs were selected, digitally photographed, and counted manually. The counting was performed three times for each photograph. The average intraepithelial TIL count for each patient was used for the statistical analysis. PD-L1 staining was assessed using the Tumor Proportion Score (TPS). TPS was defined as the number of positive tumor cells divided by the total number of viable tumor cells multiplied by 100%.

### Isolation of exosomes

Exosomes were recovered by a sequential centrifugation procedure using the Exosome Isolation Kit PS (MagCapture, Fujifilm Wako). Six milliliters of venous blood from each patient was separated into serum and cellular fractions. Cells were pelleted by centrifugation at 300×*g* for 5 min, followed by centrifugation at 1200×*g* for 20 min. To eliminate other cellular debris, the supernatant was centrifuged at 10,000×*g* for 30 min. The samples were concentrated by filtration (Vivaspin 20; Sartorius). After sample preparation, exosomes were purified using MagCapture according to the manufacturer’s instructions. Exosomes were verified by electron microscopy. The final exosome pellet was eluted with elution buffer.

### Enzyme-linked immuno-sorbent assay (ELISA) procedures

Exosome pellets isolated from 1 mL serum were resuspended using cell extraction buffer.

**Figure 1:**
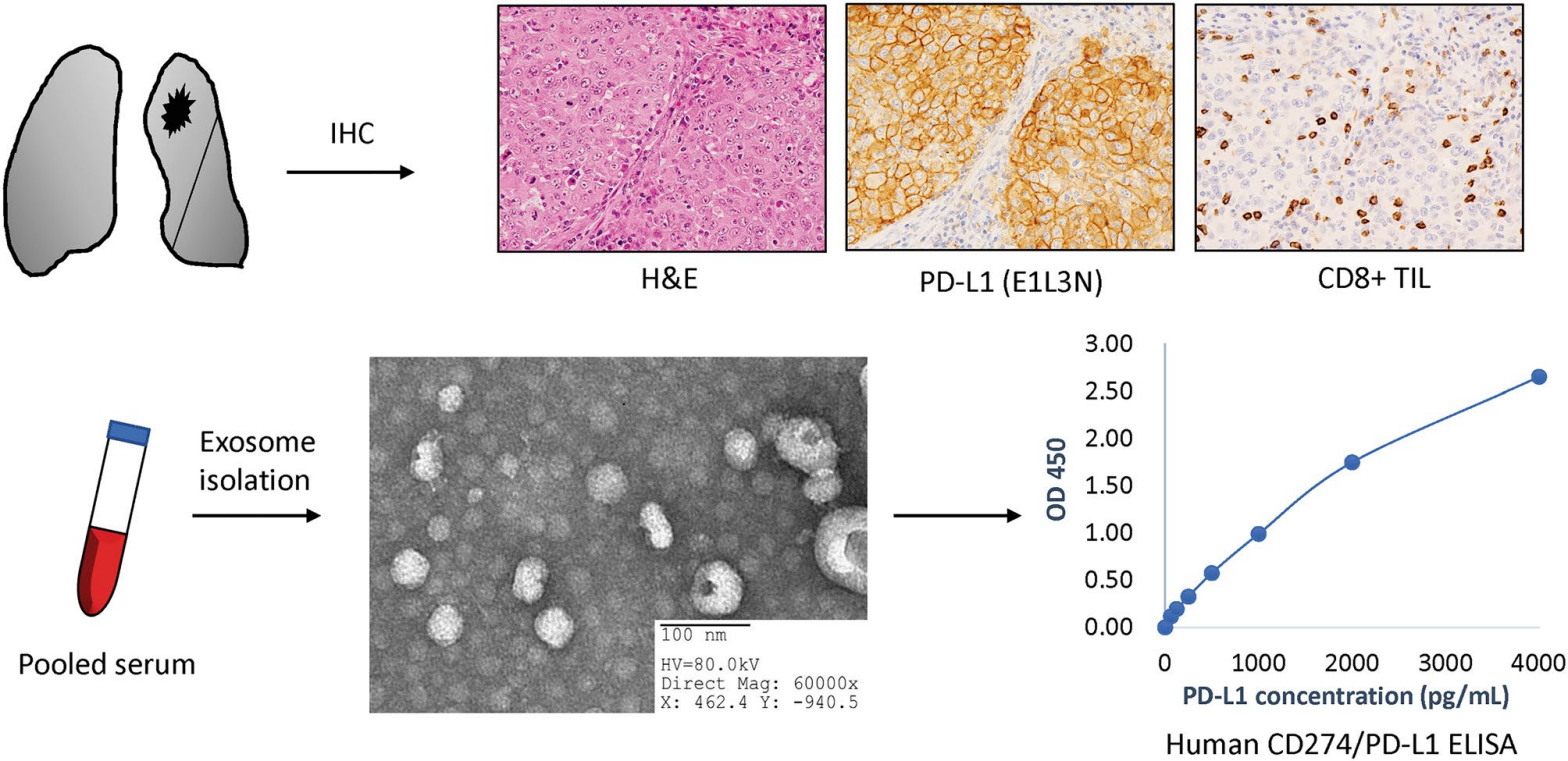
Study schema. Immunohistochemical staining for PD-L1 and CD8 was performed on whole-section samples from patients with NSCLC. Serum exosomes were isolated from patients and verified using electron microscopy. The human CD274/PD-L1 ELISA kit was used to quantify the exosomal PD-L1 concentration, and protein levels were calculated according to standard curves. *H&E* hematoxylin and eosin, *IHC* immunohistochemical staining, *PD-L1* programmed cell death-ligand 1, *CD8+ TILs* CD8^+^ tumor-infiltrating lymphocytes.

The Human CD274/PD-L1 ELISA kit (ARG81929; Arigo) was used to quantify the exosomal PD-L1 concentration following the manufacturer’s protocol. Briefly, 96-well plates with standards at different concentrations were incubated along with serum samples. After covering the antibodies, HRP-conjugated streptavidin was prepared, protected from light. Enzymatic reactions were developed, and the absorbance was measured at 450 nm using a microplate reader. Protein levels were calculated using standard curves. The study schema is shown in Fig. 1.

### Statistical analyses

Recurrence-free survival (RFS) time was measured as the interval between the date of surgery and either the date of recurrence, the date of death from any cause, or the date on which the patient was last known to be alive. RFS curves were plotted using the Kaplan–Meier method, and differences in variables were determined using the log-rank test. The Pearson chi-square test for categorical data and Student’s *t*-test for continuous data were used to compare the two groups. Pearson correlation coefficient was used to determine the correlation among serum exosomal PD-L1, tumoral PD-L1, and CD8+ TIL expression, and the value of the coefficient of determination (R) was calculated. All tests were 2-sided, and *p*-values of less than 0.05 were considered statistically significant. The SPSS statistical software package (version 26.0; DDR3 RDIMM, SPSS Inc., Chicago, IL, USA) was used for the statistical analysis.

### Ethical statement

The authors are accountable for all aspects of the work in ensuring that questions related to the accuracy or integrity of any part of the work are appropriately investigated and resolved. All procedures performed in this study involving human participants were performed in accordance with the Declaration of Helsinki (as reserved in 2013). The study was approved by the institutional review board of Tokyo Medical University (SH4064). Informed consent for the use and analysis of clinical data was obtained preoperatively for each patient.

## Results

**Figure 2:**
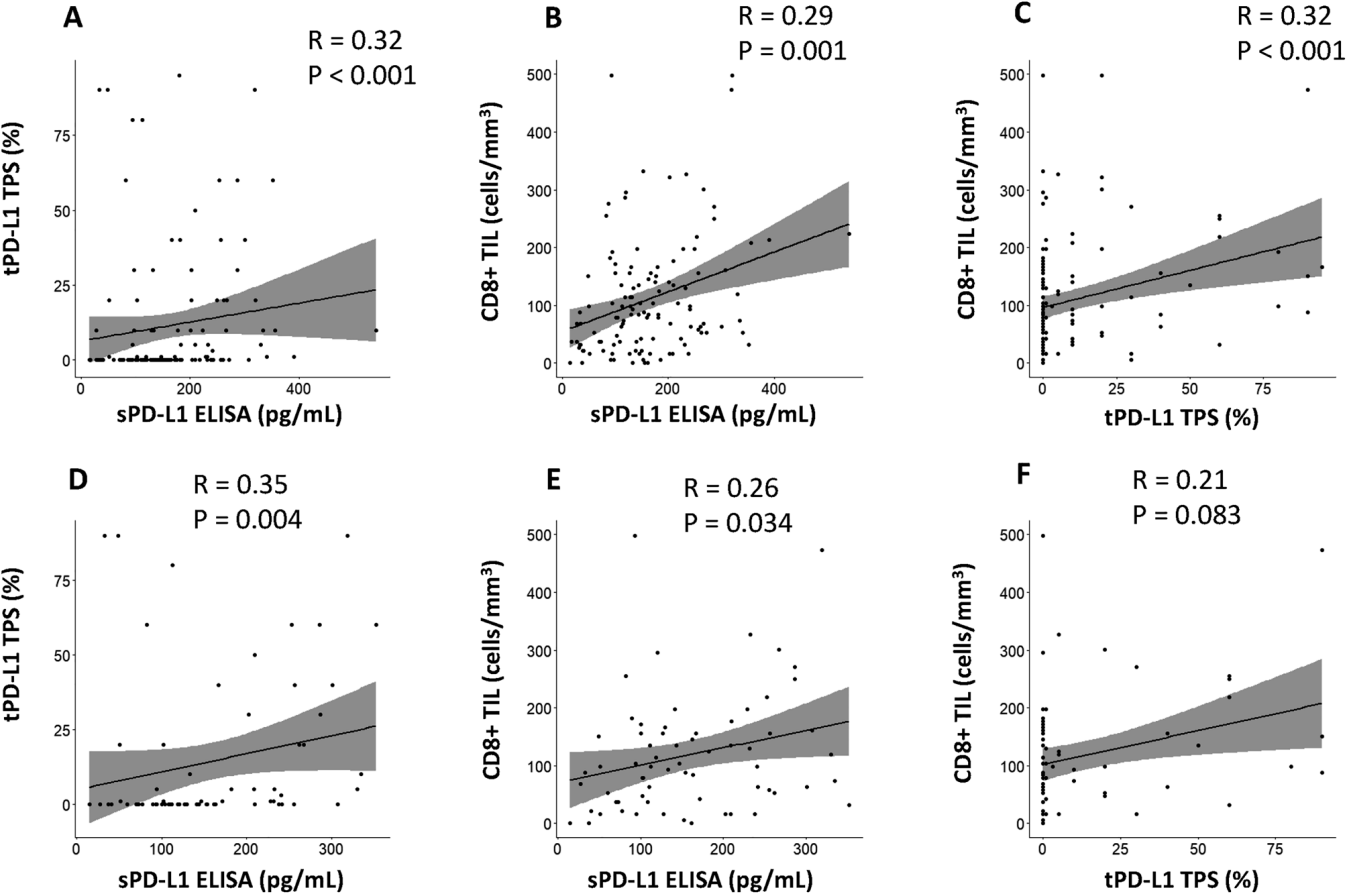
Correlative analysis of tumor PD-L1, serum exosomal PD-L1 and CD8+ TIL expression. For all patients, serum exosomal PD-L1 values correlated with tumor PD-L1 level (**A**) and the number of CD8+ TIL (**B**), while tumor PD-L1 level was associated with the number of CD8+ TIL (**C**). For 65 patients with wild type *EGFR* tumors, serum PD-L1 values correlated with tumor PD-L1 level (**D**) and the number of CD8+ TIL (**E**), while tumor PD-L1 level tended to be correlated with the number of CD8+ TIL (**F**). *sPD-L1* serum exosomal programmed cell death-ligand 1, *tPD-L1* tumor PD-L1, *CD8+ TIL* CD8^+^ tumor-infiltrating lymphocytes, *EGFR* epidermal growth factor receptor.

The study participant characteristics are summarized in Table 1. The median follow-up time for survivors was 1272 days (28–2159 days). The number of patients at pathological stage I, II and III was 65, 26 and 29, respectively. The mean values of exosomal PD-L1, tumor PD-L1, and CD8+ TILs were 164 pg/mL, 12% and 111 cells/mm^3^, respectively, while their median values were 147 pg/mL, 0%, and 83 cells/ mm^3^, respectively. Forty-one patients (34%) were found to have epidermal growth factor receptor (EGFR)-mutated lung cancer, while 65 had wild type EGFR tumors (54%); mutational status was unknown in the remaining 14 patients (12%). For all patients, serum PD-L1 levels significantly correlated with tumor PD-L1 level (R = 0.32, p < 0.001; Fig. 2A) and the number of CD8+ TILs (R = 0.29, p = 0.001; Fig. 2B); tumor PD-L1 levels were also associated with the number of CD8+ TILs (R = 0.32, p < 0.001; Fig. 2C). In all patients with wild type EGFR tumors, serum PD-L1 levels correlated with tumor PD-L1 level (R = 0.35, p = 0.004; Fig. 2D) and the number of CD8+ TILs (R = 0.26, p = 0.034; Fig. 2E), while tumor PD-L1 levels tended to be correlated with the number of CD8+ TILs (R = 0.21, p = 0.083; Fig. 2F). The receiver-operating characteristic curve of serum exosomal PD-L1 levels was created for tumor PD-L1 positivity. The area under the curve and the optimal cutoff value relevant to tumor PD-L1 positivity were 0.711 (p < 0.001) and 166 pg/mL, respectively (Supplementary Fig. S2). The relationship between various clinicopathological factors and serum/tumors PD-L1 levels showed that smokers (p = 0.024), an advanced stage (p = 0.025), non-adenocarcinoma (p = 0.001), EGFR wild-type tumors (p = 0.029), vascular invasion (p < 0.001), lymph node metastasis (p = 0.001), elevated serum PD-L1 level (p < 0.001), and an increased number of CD8+ TILs (p = 0.007) were significantly associated with patients with tumor PD-L1 ≥ 1% (Table 2). An increased number of CD8+ TILs (p = 0.014) and tumor PD-L1 positivity (p = 0.031) were significantly associated with patients with serum PD-L1 levels ≥ 166 pg/mL.

**Table 1:** Patient characteristics (n = 120).

Variables	No. of patients (%)
Age (years; mean ± SD)	41–84 (68 ± 9)
**Sex**	
Male	63 (53)
Female	57 (47)
**Smoking history**	
Yes	78 (65)
No	42 (35)
FEV_1.0_% (mean ± SD)	43.9–96.9 (72.9 ± 9.1)
Whole tumor size on chest CT (cm; mean ± SD)	0.5–9.5 (2.7 ± 1.4)
Solid tumor size on chest CT (cm; mean ± SD)	0–9.4 (2.4 ± 1.5)
**Surgical procedure**	
Sublobar resection	4 (3)
Lobectomy	116 (97)
**Pathological stage**	
I	66 (55)
II	26 (22)
III	28 (23)
**Histology**	
Adenocarcinoma	93 (78)
Non-adenocarcinoma	27 (22)
***EGFR mutation***	
Positive	41 (34)
Negative	65 (54)
Unknown	14 (12)
**Vascular invasion**	
Positive	81 (68)
Negative	39 (32)
**Lymph node metastasis**	
Positive	41 (34)
Negative	79 (66)
Exosomal PD-L1 (pg/mL; mean ± SD)	15–541 (164 ± 92)
Tumor PD-L1 (%; mean ± SD)	0–95 (12 ± 23)
CD8+ TILs (cells/mm^3^; mean ± SD)	0–499 (75 ± 52)

*SD* standard deviation, *FEV* forced expiratory volume, *CT* computed tomography, *EGFR* epidermal growth factor receptor, *PD-L1* programmed cell death-ligand 1, *CD8+ TIL* CD8^+^ tumor-infiltrating lymphocytes.

**Table 2:** Relationship between clinic-pathological variables and tumor and serum PD-L1 levels.

Variables	Tumor PD-L1 level		P*-*value	Serum PD-L1 level		P*-*value
	> 1%, n = 51 (%)	< 1%, n = 69 (%)		> 166 pg/mL, n = 49 (%)	< 166 pg/mL, n = 71 (%)	
Age (years; mean)	68	68	0.730	70	67	0.096
Sex, male	32 (63)	31 (45)	0.053	29 (59)	34 (48)	0.223
Smoking history, yes	39 (76)	39 (57)	0.024	34 (69)	44 (62)	0.402
Solid tumor size on CT (cm; mean)	2.7	2.3	0.094	2.6	2.4	0.503
p-stage, stage I	22 (43)	44 (64)	0.025	26 (53)	40 (56)	0.723
Histology, adenocarcinoma	32 (63)	61 (88)	0.001	38 (78)	55 (78)	0.991
*EGFR* mutation, positive	10 (20)	31 (45)	0.029	17 (35)	24 (34)	0.898
Vascular invasion, positive	44 (86)	37 (54)	< 0.001	36 (73)	45 (63)	0.246
Lymph node metastasis, positive	26 (51)	15 (22)	0.001	20 (41)	21 (30)	0.202
Exosomal PD-L1 (pg/mL, mean)	206	132	< 0.001	–	–	–
Tumor PD-L1 (%; mean)	–	–	–	17	8	0.031
CD8+ TIL (cells/field; mean)	140	89	0.007	139	91	0.014

*PD-L1* programmed cell death-ligand 1, *CT* computed tomography, *EGFR* epidermal growth factor receptor, *CD8+ TIL*, *CD8*^+^ tumor-infiltrating lymphocytes.

**Figure 3:**
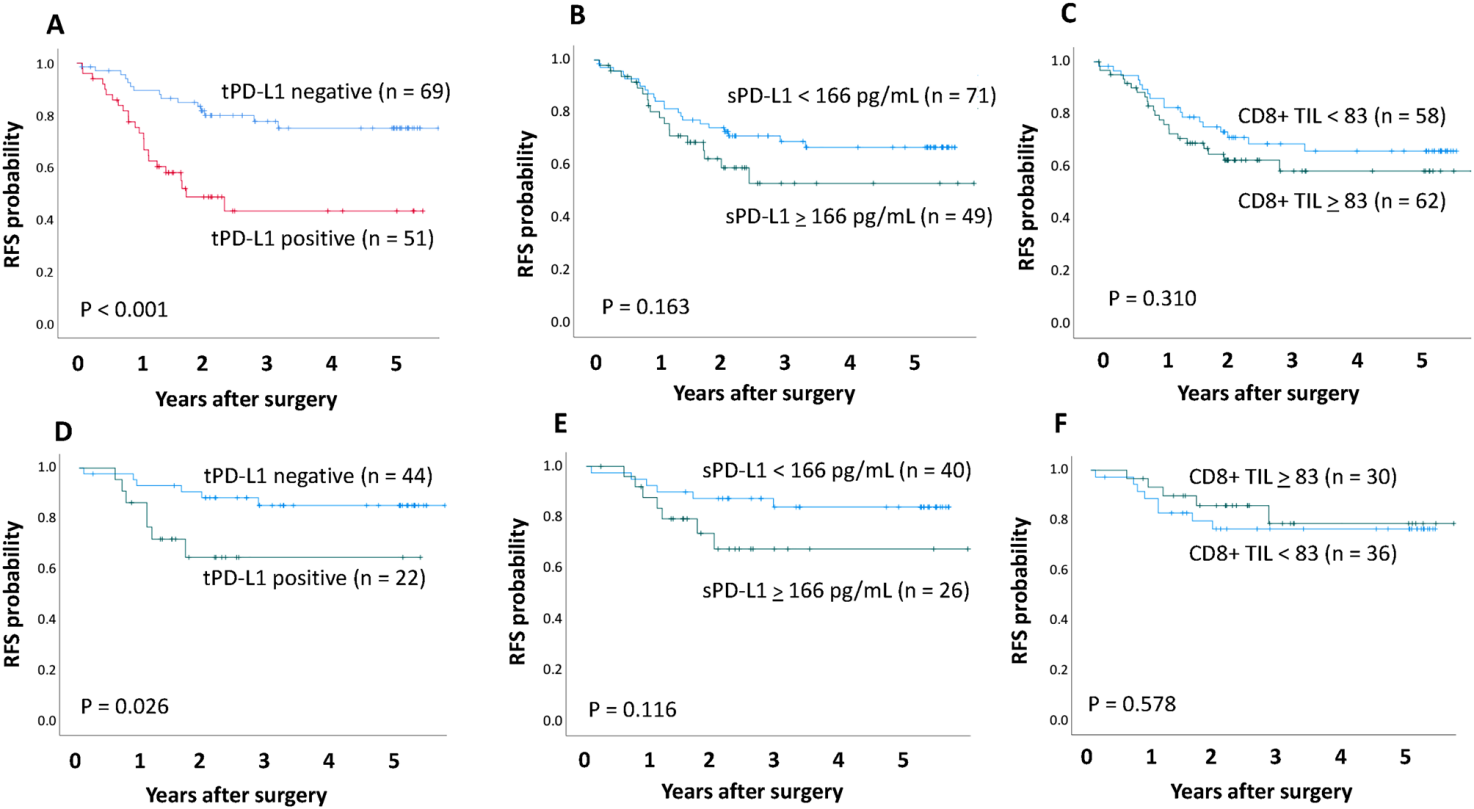
Recurrence-free survival (RFS) of the full cohorts and stage I patients according to tumor PD-L1, serum exosomal PD-L1, and CD8+ TIL status. (**A**) RFS curves of the entire population according to tumor PD-L1 (**A**), serum exosomal PD-L1 (**B**), and CD8+ TIL expression (**C**). RFS curves of patients with pathological stage I NSCLC according to tumor PD-L1 (**A**), serum exosomal PD-L1 (**B**), and CD8+ TIL expression (**C**).

**Figure 4:**
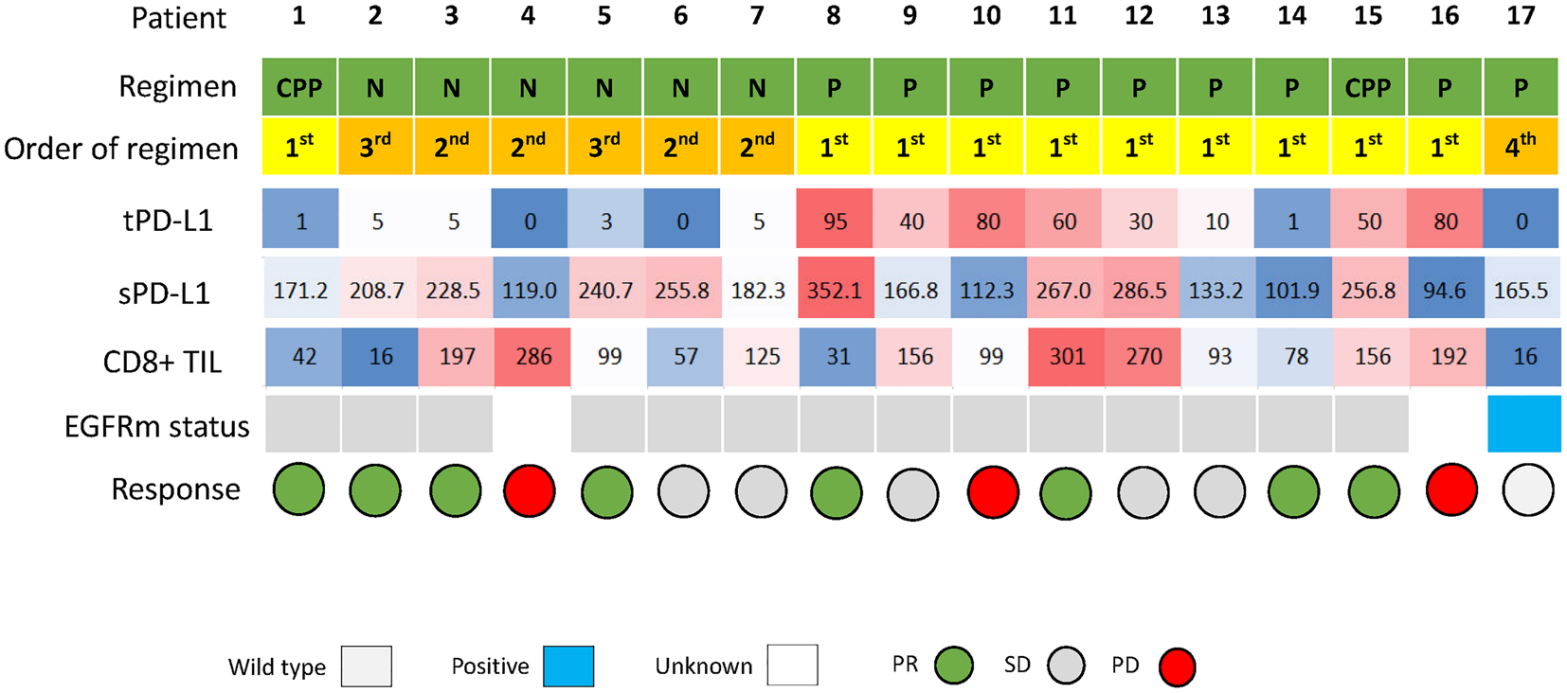
A summary of patients who were treated with PD-1 inhibitors for their recurrences. *PD-1* programmed cell death-1, *CD8+ TIL* CD8^+^ tumor-infiltrating lymphocytes, *tPD-L1* tumor PD-L1, *sPD-L1* serum exosomal PD-L1, *CPP* cisplatin + pemetrexed + pembrolizumab, *N* nivolumab, *P* pembrolizumab, *EGFRm* epidermal growth factor receptor mutation, *PR* pertial response, *SD*, stable disease, *PD* progressive disease, *TPS* tumor proportion score.

**Figure 5:**
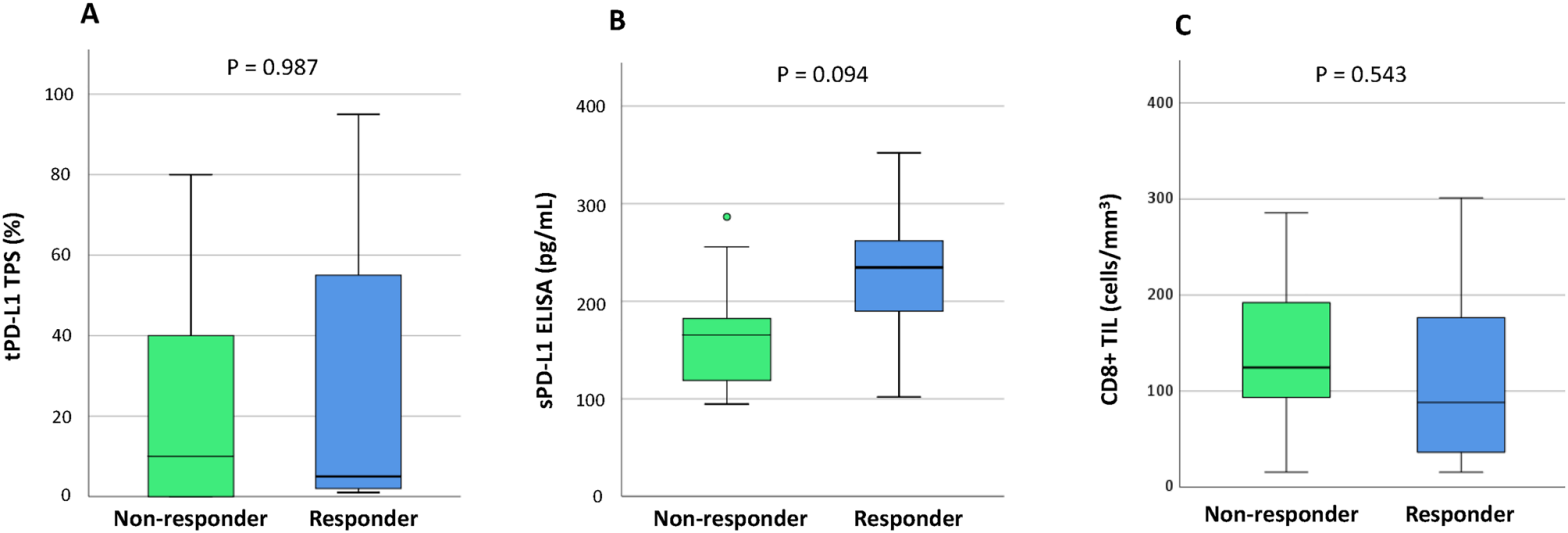
A relationship between response to PD-1 inhibitors and tumor PD-L1, serum exosomal PD-L1, CD8+ TIL levels. (**A**) A relationship between response to PD-1 inhibitors and tumor PD-L1 level. (**B**) A relationship between response to PD-1 inhibitors and serum exosomal PD-L1 level. (**C**) A relationship between response to PD-1 inhibitors and CD8+ TIL expression. *PD-1* programmed cell death-1, *CD8+ TIL* CD8^+^ tumor-infiltrating lymphocytes, *tPD-L1* tumor PD-L1, *sPD-L1* serum exosomal PD-L1.

**Figure 6:**
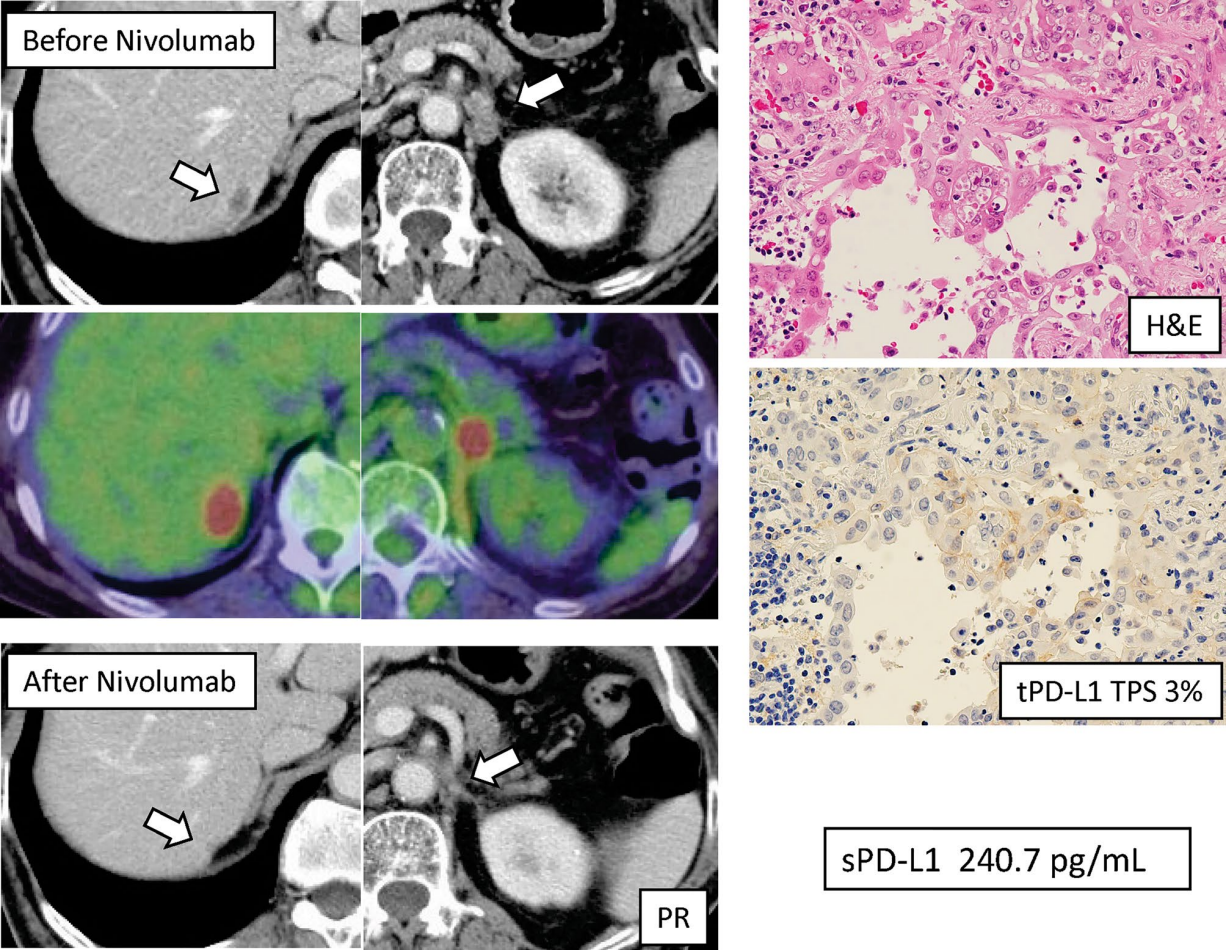
A representative patient with recurrent NSCLC showing the discrepancy between tPD-L1 and sPD-L1 level. A case with low tumor PD-L1 and high serum PD-L1 (Patient 5). Therapeutic effect of 3rd line nivolumab in this patient was partial response. *H&E* Hematoxylin and Eosin, *tPD-L1* tumor programmed cell death-ligand 1, *sPD-L1* serum exosomal PD-L1, *TPS* tumor proportion score.

RFS was significantly higher in patients with tumor PD-L1 positivity than in those with negative tumor PD-L1 (5-year RFS 75.0% vs. 43.2%, p < 0.001; Fig. 3A). Difference in RFS was observed between patients with serum PD-L1 ≥ 166 pg/mL and those with < 1660 pg/mL, but not significant (p = 0.163; Fig. 3B). According to the median value of CD8+ TILs in this study (83 cells/mm^3^), a cut-off threshold of 83 cells/ mm^3^ was set for CD8+ TILs. No statistical difference in RFS was observed between patients with CD8+ TILs ≥ 83 cells/mm^3^ and those with < 83 cells/mm^3^ (p = 0.310; Fig. 3C). For 66 pathological stage I patients, tumor PD-L1 status was significantly associated with RFS (p = 0.026; Fig. 3D), and serum exosomal PD-L1 also tended to be associated with RFS (p = 0.116; Fig. 3E). There was no statistical difference in RFS between EGFR-negative patients with CD8+ TILs ≥ 83 cells/mm^3^ and those with < 83 cells/mm^3^ (p = 0.578; Fig. 3F). During the follow-up period, 17 patients underwent postoperative recurrence and anti-PD-1 treatment. A list of patients who received PD-1inhibitors for recurrent disease is shown in (Supplementary Table S1). There were ten adenocarcinomas, four squamous cell carcinomas, and three other types of histology. The time to recurrence from surgery ranged from 5 to 29 months. The type of PD-1 inhibitors included nine pembrolizumab monotherapies, six nivolumab monotherapies, and two combination immunotherapy regimens. The results of the values of tumor PD-L1, serum exosomal PD-L1, and CD8+ TILs, and the therapeutic effects in patients undergoing PD-1 inhibitors for recurrent diseases are summarized in Fig. 4. Box plots are used to visualize the comparative distributions of tumor PD-L1 (Fig. 5A), serum exosomal PD-L1 (Fig. 5B), and CD8+ TILs (Fig. 5C) to analyze their association with response. Although no significant correlations were observed, serum exosomal PD-L1 level showed the highest correlation (p = 0.094); all the patients with serum exosomal PD-L1 ≥ 166 pg/mL demonstrated a disease control to PD-1 inhibitors. A representative case of PD-L1 inhibitor presenting the discrepancy between tumor PD-L1 and serum exosomal PD-L1 level is shown in Fig. 6; this patient demonstrated low tumor PD-L1 (TPS 3%) and high serum exosomal PD-L1 (240 pg/mL; patient 5 in Supplementary Table S1). The therapeutic effect of 3^rd^ line nivolumab in this patient was found to be a partial response.

## Discussion

In the present study, it was found that serum exosomal PD-L1 levels were useful for predicting anti-PD-1 therapies for recurrent NSCLC, and that they tended to be associated with survival in patients with NSCLC. Although serum exosomal PD-L1 expression was significantly associated with CD8+ TILs and tumor PD-L1 levels, no correlation was observed between the expression and pathological stage, lymph node status, or *EGFR* mutation status. When comparing tumor PD-L1 and serum exosomal PD-L1 expression, there were several conflicting recurrent cases showing low tumor PD-L1, and high serum PD-L1, and vice versa.

Antibodies targeting PD-1/PD-L1 pathways have emerged as the gold-standard treatment for first- or second-line treatment of stage IV and recurrent NSCLC^1–5^. PD-L1 expression assessed by IHC staining has been widely evaluated in clinical trials as a predictive biomarker^1–6^. Patients using pembrolizumab monotherapy for previously treated PD-L1-positive NSCLC experienced have been found to have prolonged overall survival (OS) compared to those treated with docetaxel^2^. Pembrolizumab also significantly improved progression-free survival and OS compared with platinum-based chemotherapy in patients with chemo-naïve advanced NSCLC who had a PD-L1 TPS of 50% or greater and did not have *EGFR* mutation or anaplastic lymphoma kinase (*ALK*) rearrangement^3,5^. Moreover, the Keynote-042 trial showed that the therapeutic effect of pembrolizumab remained significant in those with a TPS of 1–49%^4^. However, PD-L1 expression is known to be highly heterogeneous with a low interobserver and inter-assay reproducibility, and discordance due to different antibodies, limited specificity, different platforms, and different thresholds also exists^22–25^. Because of this, there is a need to identify and develop other effective markers for clinical use.

TILs are reportedly associated with treatment effects of ICIs^8,25–27^. In particular, CD8+ TILs are thought to play a pivotal role in directly killing tumor cells as well as maintaining immune surveillance; these functions could be prevented by the signaling produced by the PD-1/PD-L1 axis^25,28^. Many studies have also demonstrated that CD8+ TIL levels are a significant prognostic factor in advanced and early-stage lung cancer^7–9,29,30^. Previous studies demonstrated that the frequency and prognostic impact of TILs differed according to tumor histology and staging, and that the presence of TILs was associated with improved survival exclusively in non-adenocarcinomas^29,31^. In the present study, no significant differences were found in RFS and anti-PD-1 responses according to the expression of CD8+ TILs. This could be because of a large proportion of adenocarcinoma cases and/or a highly heterogeneous population in terms of pathological stage.

In the current study, tumor PD-L1 expression status was significantly associated with prognosis in pathological stage I and stage I–III NSCLC. Numerous published studies have demonstrated the prognostic significance of PD-L1 expression in NSCLC^32–37^. However, some studies have shown that high PD-L1 expression in tumors is a favorable prognostic biomarker for survival, whereas currently no tumor PD-L1 status is a factor associated with favorable outcomes^32–40^. D’Arcangelo et al. state that a possible reason for this discrepancy was patient ethnicity, pointing out that the only studies with a negative prognostic impact of PD-L1 expression were those conducted in the Asian population^36^. The observed high frequency of *EGFR* mutations in Asian patients compared with Caucasian populations is a well-known, and NSCLC harboring *EGFR* mutations or *ALK* rearrangements are reported to be associated with low efficacy of ICIs^39,41–44^. Different antibodies used for IHC staining, different experimental platforms, different PD-L1 thresholds, and different tumor biology according to ethnicity might be responsible for these conflicting prognostic results.

PD-L1 is expressed on tumor cells, immune cells, and other cells in the tumor microenvironment, as well as being found in extracellular forms such as exosomes^18–21^. Recent studies have shown that PD-L1 expressing exosomes can inhibit antitumor immune responses either locally and systemically, depending on the target cell's location^18,19,21^. Del Re et al. studied the association between PD-L1 mRNA in plasma-derived exosomes and response to anti-PD-1 treatments in patients with melanoma and NSCLC^45^. The pre-treatment exosomal PD-L1 mRNA level was significantly higher in responders than non-responders, consistent with our results, and exosomal PD-L1 levels significantly decreased after anti-PD-1 treatments in responders but not in non-responders^45^. Exosomes are involved in the immune escape, and we hypothesized that exosomes highly expressing PD-L1 to communicate tumor inhibitory signals to effector cells such as NK cells, macrophages, dendritic cells, and T-cells may contribute to an increased response to anti-PD-1 treatments. Further research with larger cohorts of patients is warranted, and it is vital to determine whether serum exosomal PD-L1 and tumor PD-L1 status or other blood biomarkers can be used as a more accurate method for predicting the efficacy of anti-PD-1/PD-L1 therapies.

Exosomal PD-L1 has been shown to be a poor prognostic marker in patients with gastric cancer and head and neck cancer^46,47^. In the current study, serum exosomal PD-L1 status tended to be associated with RFS both in the entire cohort and in p-stage I cohorts. An experimental study showed that exosomal PD-L1 enables cancer cells to evade anti-tumor immunity, and genetic blockade of exosomal PD-L1 extends survival in mice by promoting anti-tumor immunity^19^. Further large-sampled studies are needed to validate the clinical potential of exosomal PD-L1.

Despites its insights, this study is limited by its retrospective nature and potential biases. The cut-off value of serum exosomal PD-L1 of 166 pg/mL, which dichotomized the high and low groups is somewhat arbitrary. The number of patients in this study and the number of recurrent NSCLC cases were also too small to provide strong statistical power for the drawn conclusions. There was also heterogeneity in the type of anti-PD-1 therapies used for recurrent diseases as well as in the order of regimens, and the isolation and characterization methods used for exosome study are also still a matter of debate. Further studies are required before this liquid biopsy approach for patients who require ICIs can be proven effective and successfully applied in daily clinical practice.

In the present study, our findings demonstrated that the measurement of serum exosomal PD-L1 as a quantitative complementary factor together with tumor PD-L1 status might help predict anti-PD-1 response and assess clinical outcomes in patients with NSCLC. This blood-based liquid biopsy approach can contribute to the appropriate ICI treatment decision-making process in both advanced and recurrent lung cancer when there is a limit to the amount of tissue that can be harvested.

## Supplementary Information

Supplementary Legends.

Supplementary Figure S1.

Supplementary Figure S2.

Supplementary Table S1.

## Abbreviations

NSCLC: Non-small cell lung caner
PD-1: Programmed cell death-1
PD-L1: Programmed cell death ligand 1
CD8+ TIL: CD8^+^ tumor-infiltrating lymphocyte
ICI: Immune checkpoint inhibitor
IHC: Immunohistochemical
ELISA: Enzyme-linked immuno-sorbent assay
EGFR: Epidermal growth factor receptor
ALK: Anaplastic lymphoma kinase
TMB: Tumor mutation burden
RFS: Recurrence-free survival
CT: Computed tomography
PR: Partial response
SD: Stable disease
PD: Progressive disease

## References

[1] Garon EB, Rizvi NA, Hui R (2015). Pembrolizumab for the treatment of non-small-cell lung cancer. N. Engl. J. Med..

[2] Herbst RS, Baas P, Kim DW (2016). Pembrolizumab versus docetaxel for previously treated, PD-L1-positive, advanced non-small-cell lung cancer (KEYNOTE-010): A randomised controlled trial. Lancet.

[3] Reck M, Rodriguez-Abreu D, Robinson AG (2016). Pembrolizumab versus chemotherapy for PD-L1-positive non-small-cell lung cancer. N. Engl. J. Med..

[4] Mok TSK, Wu YL, Kudaba I (2019). Pembrolizumab versus chemotherapy for previously untreated, PD-L1-expressing, locally advanced or metastatic non-small-cell lung cancer (KEYNOTE-042): A randomised, open-label, controlled, phase 3 trial. Lancet.

[5] Reck M, Rodriguez-Abreu D, Robinson AG (2019). Updated analysis of KEYNOTE-024: Pembrolizumab versus Platinum-based chemotherapy for advanced non-small-cell lung cancer with PD-L1 tumor proportion score of 50% or greater. J. Clin. Oncol..

[6] Yarchoan M, Albacker LA, Hopkins AC (2019). PD-L1 expression and tumor mutational burden are independent biomarkers in most cancers. JCI Insight..

[7] Donnem T, Hald SM, Paulsen EE (2015). Stromal CD8+ T-cell density-a promising supplement to TNM Staging in non-small cell lung cancer. Clin. Cancer Res..

[8] Teng F, Meng X, Wang X (2016). Expressions of CD8+TILs, PD-L1 and Foxp3+TILs in stage I NSCLC guiding adjuvant chemotherapy decisions. Oncotarget.

[9] Fumet JD, Richard C, Ledys F (2018). Prognostic and predictive role of CD8 and PD-L1 determination in lung tumor tissue of patients under anti-PD-1 therapy. Br. J. Cancer.

[10] Bach DH, Hong JY, Park HJ, Lee SK (2017). The role of exosomes and miRNAs in drug-resistance of cancer cells. Int. J. Cancer.

[11] Guo W, Gao Y, Li N (2017). Exosomes: New players in cancer (review). Oncol. Rep..

[12] Vanni I, Alama A, Grossi F (2017). Exosomes: A new horizon in lung cancer. Drug Discov. Today.

[13] Wen SW, Sceneay J, Lima LG (2016). The biodistribution and immune suppressive effects of breast cancer-derived exosomes. Cancer Res..

[14] Peinado H, Aleckovic M, Lavotshkin S (2012). Melanoma exosomes educate bone marrow progenitor cells toward a pro-metastatic phenotype through MET. Nat. Med..

[15] Thery C, Zitvogel L, Amigorena S (2002). Exosomes: Composition, biogenesis and function. Nat. Rev. Immunol..

[16] Kahlert C, Kalluri R (2013). Exosomes in tumor microenvironment influence cancer progression and metastasis. J. Mol. Med. (Berl.).

[17] Costa-Silva B, Aiello NM, Ocean AJ (2015). Pancreatic cancer exosomes initiate pre-metastatic niche formation in the liver. Nat. Cell. Biol..

[18] Daassi D, Mahoney KM, Freeman GJ (2020). The importance of exosomal PDL1 in tumour immune evasion. Nat. Rev. Immunol..

[19] Poggio M, Hu T, Pai CC (2019). Suppression of exosomal PD-L1 induces systemic anti-tumor immunity and memory. Cell.

[20] Li C, Li C, Zhi C (2019). Clinical significance of PD-L1 expression in serum-derived exosomes in NSCLC patients. J. Transl. Med..

[21] Chen G, Huang AC, Zhang W (2018). Exosomal PD-L1 contributes to immunosuppression and is associated with anti-PD-1 response. Nature.

[22] McLaughlin J, Han G, Schalper KA (2016). Quantitative assessment of the heterogeneity of PD-L1 expression in non-small-cell lung cancer. JAMA Oncol..

[23] Greillier L, Tomasini P, Barlesi F (2018). The clinical utility of tumor mutational burden in non-small cell lung cancer. Transl. Lung Cancer Res..

[24] Bassanelli M, Sioletic S, Martini M (2018). Heterogeneity of PD-L1 expression and relationship with biology of NSCLC. Anticancer Res..

[25] Yi M, Jiao D, Xu H (2018). Biomarkers for predicting efficacy of PD-1/PD-L1 inhibitors. Mol. Cancer.

[26] Teng MW, Ngiow SF, Ribas A (2015). Classifying cancers based on T-cell infiltration and PD-L1. Cancer Res..

[27] Tomioka N, Azuma M, Ikarashi M (2018). The therapeutic candidate for immune checkpoint inhibitors elucidated by the status of tumor-infiltrating lymphocytes (TILs) and programmed death ligand 1 (PD-L1) expression in triple negative breast cancer (TNBC). Breast Cancer.

[28] Solomon B, Young RJ, Bressel M (2018). Prognostic significance of PD-L1(+) and CD8(+) immune cells in HPV(+) oropharyngeal squamous cell carcinoma. Cancer Immunol. Res..

[29] Kinoshita T, Muramatsu R, Fujita T (2016). Prognostic value of tumor-infiltrating lymphocytes differs depending on histological type and smoking habit in completely resected non-small-cell lung cancer. Ann. Oncol..

[30] Shimizu K, Okita R, Saisho S (2019). Comparative study of the PD-L1 expression and CD8+ tumor-infiltrating lymphocyte between surgically resected and matched re-biopsy specimens in recurrent non-small cell lung cancer. Ther. Clin. Risk Manage..

[31] Ruffini E, Asioli S, Filosso PL (2009). Clinical significance of tumor-infiltrating lymphocytes in lung neoplasms. Ann. Thorac. Surg..

[32] Igawa S, Sato Y, Ryuge S (2017). Impact of PD-L1 expression in patients with surgically resected non-small-cell lung cancer. Oncology.

[33] Takada K, Okamoto T, Toyokawa G (2017). The expression of PD-L1 protein as a prognostic factor in lung squamous cell carcinoma. Lung Cancer.

[34] Tsao MS, Le Teuff G, Shepherd FA (2017). PD-L1 protein expression assessed by immunohistochemistry is neither prognostic nor predictive of benefit from adjuvant chemotherapy in resected non-small cell lung cancer. Ann. Oncol..

[35] Zhou C, Tang J, Sun H (2017). PD-L1 expression as poor prognostic factor in patients with non-squamous non-small cell lung cancer. Oncotarget.

[36] D'Arcangelo M, D'Incecco A, Ligorio C (2019). Programmed death ligand 1 expression in early stage, resectable non-small cell lung cancer. Oncotarget.

[37] Cooper WA, Tran T, Vilain RE (2015). PD-L1 expression is a favorable prognostic factor in early stage non-small cell carcinoma. Lung Cancer.

[38] Jin Y, Shen X, Pan Y (2019). Correlation between PD-L1 expression and clinicopathological characteristics of non-small cell lung cancer: A real-world study of a large Chinese cohort. J. Thorac. Dis..

[39] Miyawaki E, Murakami H, Mori K (2020). PD-L1 expression and response to pembrolizumab in patients with EGFR-mutant non-small cell lung cancer. Jpn. J. Clin. Oncol..

[40] Tashima Y, Kuwata T, Yoneda K (2020). Prognostic impact of PD-L1 expression in correlation with neutrophil-to-lymphocyte ratio in squamous cell carcinoma of the lung. Sci. Rep..

[41] Shi Y, Au JS, Thongprasert S (2014). A prospective, molecular epidemiology study of EGFR mutations in Asian patients with advanced non-small-cell lung cancer of adenocarcinoma histology (PIONEER). J. Thorac. Oncol..

[42] Chougule A, Prabhash K, Noronha V (2013). Frequency of EGFR mutations in 907 lung adenocarcioma patients of Indian ethnicity. PLoS ONE.

[43] Lisberg A, Cummings A, Goldman JW (2018). A phase II study of pembrolizumab in EGFR-mutant, PD-L1+, tyrosine kinase ihibitor naive patients with advanced NSCLC. J. Thorac. Oncol..

[44] Gainor JF, Shaw AT, Sequist LV (2016). EGFR mutations and ALK rearrangements are associated with low response rates to PD-1 pathway blockade in non-small cell lung cancer: A retrospective analysis. Clin. Cancer Res..

[45] Del Re M, Marconcini R, Pasquini G (2018). PD-L1 mRNA expression in plasma-derived exosomes is associated with response to anti-PD-1 antibodies in melanoma and NSCLC. Br. J. Cancer.

[46] Theodoraki MN, Yerneni SS, Hoffmann TK (2018). Clinical significance of PD-L1(+) exosomes in plasma of head and neck cancer patients. Clin. Cancer Res..

[47] Fan Y, Che X, Qu J (2019). Exosomal PD-L1 retains immunosuppressive activity and is associated with gastric cancer prognosis. Ann. Surg. Oncol..

