# Supplementary material for: Serum-derived exosomal PD-L1 expression to predict anti-PD-1 response and in patients with non-small cell lung cancer
Source: Sci Rep. 2021 Apr 9;11:7830. doi: 10.1038/s41598-021-87575-3 (PMC8035184; doi:10.1038/s41598-021-87575-3)
Supplement: Supplementary file 1 — Supplementary Legends. [file 41598_2021_87575_MOESM1_ESM.docx]

**Supplementary Figure 1**

Consort diagram of patients included in this study.

**Supplementary Figure 2**

Receiver-operating characteristics area under the curve (AUC) (0.711, 95% confidence interval, 0.612 – 0.809, P < 0.001) for serum exosomal PD-L1 level to identify tumor PD-L1 expression.
