# Supplementary figures and images for: Serum-derived exosomal PD-L1 expression to predict anti-PD-1 response and in patients with non-small cell lung cancer
Source: Sci Rep. 2021 Apr 9;11:7830. doi: 10.1038/s41598-021-87575-3 (PMC8035184; doi:10.1038/s41598-021-87575-3)

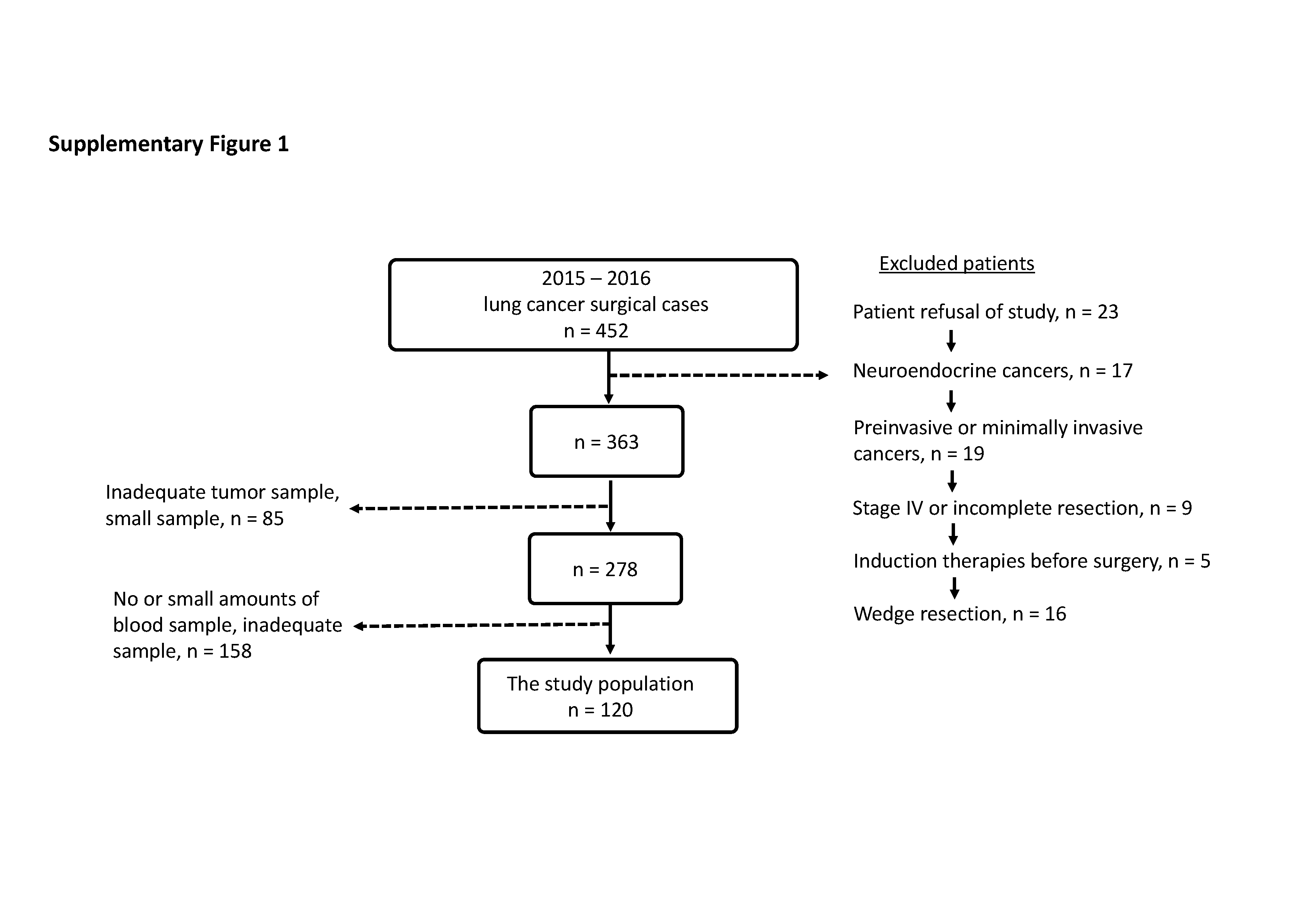

Supplement: Supplementary file 2 — Supplementary Figure S1. [file 41598_2021_87575_MOESM2_ESM.tif]

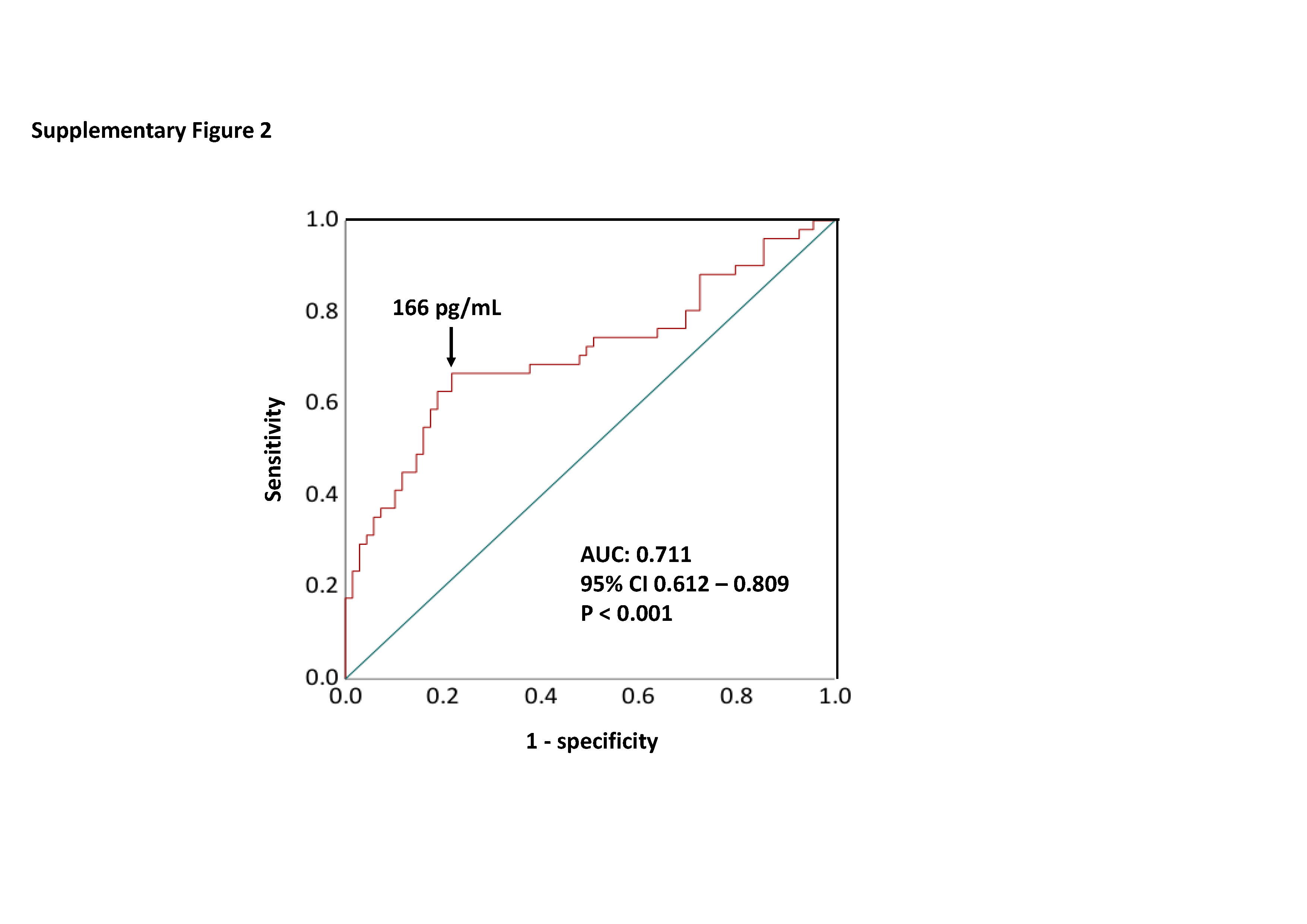

Supplement: Supplementary file 3 — Supplementary Figure S2. [file 41598_2021_87575_MOESM3_ESM.tif]
