# Supplementary material for: Serum-derived exosomal PD-L1 expression to predict anti-PD-1 response and in patients with non-small cell lung cancer
Source: Sci Rep. 2021 Apr 9;11:7830. doi: 10.1038/s41598-021-87575-3 (PMC8035184; doi:10.1038/s41598-021-87575-3)
Supplement: Supplementary file 4 — Supplementary Table S1. [file 41598_2021_87575_MOESM4_ESM.docx]

Supplementary Table 1 Patients who received PD-1 inhibitors for postoperative recurrent diseases

| No. | Stage | Histology | Time to recurrence | Recurrence pattern | PD-1 inhibitors | Order of regimen | Clinical response |
| --- | --- | --- | --- | --- | --- | --- | --- |
| 1 | IIB | Adeno | 29 months | Both | Cisplatin  Pemetrexed  Pembrolizumab | 1^st^ | PR |
| 2 | IB | Adeno | 9 months | Local | Nivolumab | 3^rd^ | PR |
| 3 | IIB | Adeno | 10 months | Local | Nivolumab | 2^nd^ | PR |
| 4 | IIIA | Squamous | 9 months | Distant | Nivolumab | 2^nd^ | PD |
| 5 | IIA | Pleomorphic | 8 months | Distant | Nivolumab | 3^rd^ | PR |
| 6 | IB | Adeno | 10 months | Distant | Nivolumab | 2^nd^ | SD |
| 7 | IIIA | Adeno | 10 months | Local | Nivolumab | 2^nd^ | SD |
| 8 | IIIA | Adeno | 5 months | Both | Pembrolizumab | 1^st^ | PR |
| 9 | IIIA | Adsq | 17 months | Local | Pembrolizumab | 1^st^ | SD |
| 10 | IIB | Pleomorphic | 11 months | Distant | Pembrolizumab | 1^st^ | PD |
| 11 | IA | Squamous | 14 months | Local | Pembrolizumab | 1^st^ | PR |
| 12 | IIIB | Adeno | 14 months | Local | Pembrolizumab | 1^st^ | SD |
| 13 | IIB | Squamous | 6 months | Distant | Pembrolizumab | 1^st^ | PD |
| 14 | IIIA | Adeno | 10 months | Distant | Pembrolizumab | 1^st^ | PR |
| 15 | IB | Adeno | 7 months | Local | Cisplatin  Pemetrexed  Pembrolizumab | 1^st^ | PR |
| 16 | IIA | Squamous | 21 months | Local | Pembrolizumab | 1^st^ | PD |
| 17 | IIB | Adeno | 4 months | Local | Pembrolizumab | 4^th^ | PD |

PD-1, programmed cell death-1; Adsq, adenosquamous cell carcinoma; PR, partial response; PD, progressive disease; SD, stable disease.
